# 2D/2D Bi_2_Se_3_/SnSe_2_ heterostructure with rapid NO_2_ gas detection

**DOI:** 10.3389/fchem.2024.1425693

**Published:** 2024-07-26

**Authors:** Shuangshuang Yi, Cunguang Chen, Meiling Yu, Juanjuan Hao, You Wang

**Affiliations:** School of Materials Science and Engineering, Harbin Institute of Technology, Harbin, China

**Keywords:** 2D, NO_2_ Sensing, heterostructure, room-temperature, flexible sensor

## Abstract

Heterostructure engineering is crucial for enhancing gas sensing performance. However, achieving rapid response for room-temperature NO_2_ sensing through rational heterostructure design remains a challenge. In this study, a Bi_2_Se_3_/SnSe_2_ 2D/2D heterostructure was synthesized by hydrothermal method for the rapid detection of NO_2_ at room temperature. By combining Bi_2_Se_3_ nanosheets with SnSe_2_ nanosheets, the Bi_2_Se_3_/SnSe_2_ sensor demonstrated and the lowest detection limit for NO_2_ a short response time (15 s) to 10 ppm NO_2_ at room temperature, reaches 25 ppb. Furthermore the sensor demonstrates significantly larger response to NO_2_ than to other interfering gases, including 10 ppm NO_2_, H_2_S, NH_3_, CH_4_, CO, and SO_2_,demonstrating its outstanding selectivity. And we discuss the mechanism of related performance enhancement.

## 1 Introduction

Flexible sensors for pressure, temperature, humidity, strain, and heat flux have garnered significant attention in the fields of electronic skin, environmental monitoring, and body temperature monitoring (Ansari et al., 2023). Gas sensing has become a critical aspect in atmospheric environment monitoring, industrial production, and medical healthcare applications (Yang et al., 2023).NO_2_ stands as one of the most prevalent air pollutants. Studies indicate that even brief exposure to concentrations as low as 3 ppm can cause eye and lung irritation in human, while also compromising respiratory infection resistance, potentially resulting in fatality in severe cases (Spirtas et al., 1986; Qureshi et al., 2003; Rathi and Pal 2020).Therefore, the development of highly responsive and rapid gas sensing technologies for the detection of low-concentration NO_2_ at routine temperatures assumes paramount importance in practical applications.

Two-dimensional metal chalcogenides (TMDs) have attracted considerable interest in gas sensing owing to their superior electrical properties, large surface area, and abundant adsorption sites (Zheng et al., 2016; Sardana et al., 2022; Yang et al., 2022; Wang et al., 2023; Yang et al., 2024).Huang et al. modified two-dimensional material MoS_2_ with zinc oxide nanoparticles to improve the response of MoS_2_ to NO_2_ (Han et al., 2018).While tin (Sn) is not classified as a transition metal, its selenide compound can adopt a 2D-layered hexagonal structure similar to that of TMDs (D'Olimpio et al., 2022; Harish and Sathyakam, 2022). Moreover heterostructures offer significant potential for a broad applications, including high-speed electronics and optoelectronic devices. By integrating SnS_2_ nanosheets with the Ti_3_C_2_T_x_ skeleton platform, Han et al. achieved a good response to 50ppm triethylamine at room temperature (Han et al., 2023). So the fabrication of heterostructures has been widely utilized to further enhance the sensing performance of gas sensors based on SnSe_2_. For instance, Li et al. fabricated a chemiresistive sensor based on a SnSe_2_/SnO_2_ heterojunction using the thermal oxidation method for the detection of NO_2_ gas (Li et al., 2020). While significant advancements have been achieved in enhancing the sensing capabilities of SnSe_2_ for NO_2_ detection, there is still a need for further improvement, particularly in terms of reducing the response time, as well as improving selectivity. An important limitation of two-dimensional materials for room temperature gas sensing is the long response recovery time due to the slow desorption process. It is well understood that in comparison to 0D/2D and 1D/2D heterostructures, 2D/2D heterostructures offer closer interfacial contacts, facilitating enhanced charge transfer and thereby improving sensing properties. It is anticipated that the development of these 2D/2D heterostructures on SnSe_2_ could efficiently accelerate the response time.

Enhancing selectivity is a critical factor for gas sensing equipment. Current strategies employed to improve the selectivity of gas sensing devices encompass precious metal modification, heterostructural construction, ultraviolet irradiation, and external auxiliary heatin (Majhi et al., 2015; Zhang et al., 2016; Joshi et al., 2018) Bi_2_Se_3_, a representative 2D semiconductor, has gained significant interest across various fields like optoelectronic circuit, photocatalysis, and batteries due to its favorable bandgap, exceptional stability, and ease of synthesis (Huang et al., 2020; Li et al., 2022). Moreover, the lower work function of Bi_2_Se_3_ compared to SnSe_2_ promotes facilitates electron migration from Bi_2_Se_3_ to SnSe_2_, resulting in a decrease in the work function of SnSe_2_. This reduced work function of SnSe_2_ facilitates electron transfer to NO_2_ molecules during sensing (Li et al., 2024). Additionally, the remarkable catalytic properties of Bi_2_Se_3_ contribute to lowering the activation energy needed for gas sensing, ultimately enhancing sensing performance, particularly in reducing response time and increasing selectivity. Consequently, the engineering of 2D/2D heterostructures by combining Bi_2_Se_3_ nanosheets and SnSe_2_ holds promise for developing sensors with improved sensitivity and rapid response at room temperatures.

In this study, 2D/2D heterostructures of SnSe_2_/Bi_2_Se_3_ were synthesized using a combination of colloidal method and solvothermal method with a laminated stack structure. The optimized SnSe_2_/Bi_2_Se_3_ sensor demonstrated a notable reduction in response time for 10 ppm NO_2_, dropping from 73 to 15 s, and a decrease in recovery time from 300 to 110 s. The optimized sensor also exhibits a high response to NO_2_, with a high response rate of 130% to 10ppm of NO_2_. These improved sensing capabilities can be ascribed to enhanced transfer of charges and the larger number of adsorption sites, which are both enabled by the SnSe_2_/Bi_2_Se_3_ 2D/2D heterostructures. Additionally, the fatigue tests of the sensor after 100, 500, and 1,000 cycles of bending and relaxation showed no significant decrease in response values, demonstrating its excellent mechanical performance. These findings present a novel and effective strategy for developing a practical NO_2_ gas sensor based on SnSe_2_.

## 2 Materials and methods

### 2.1 Synthesis of SnSe_2_ nanoplates

Firstly, 0.5 mmol SnCl_4_·5H_2_O, 1.5 mmol SeO_2_, and 0.5 mmol 1, 10 -phenanthroline were dissolved in a three-neck flask. The mixture was stirred for 10 min under a highly pure N_2_ atmosphere at room temperature. Following a 10-min N_2_ degassing, the solution underwent heating to 110 °C while being stirred magnetically for an additional 10 min. Then, further increased to 260 °C and maintained for another 30 min with continuous stirring. Cooling down to room temperature, the resulting products were collected and centrifuged under three rounds of washing: first with cyclohexane and then with ethanol. Ultimately, the products were kept at 70°C for 10 h.

### 2.2 Synthesis of Bi_2_Se_3_/SnSe_2_ heterostructures

**FIGURE 1:**
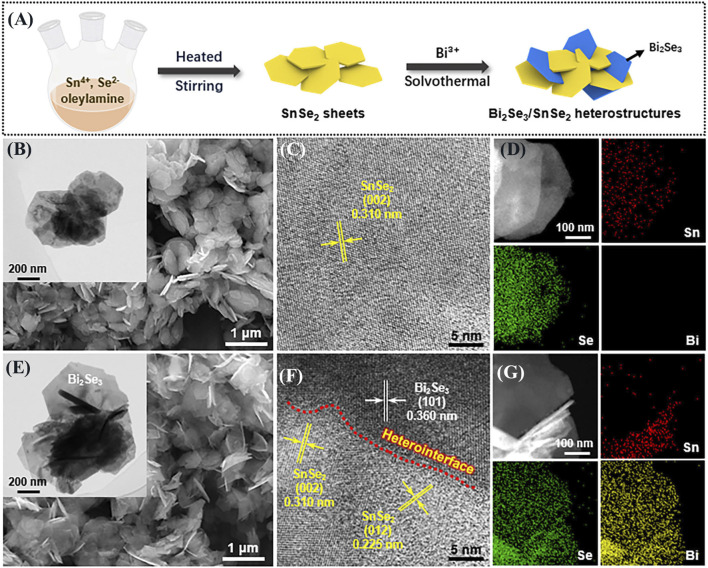
**(A)** Diagram showing the synthetic process of Bi_2_Se_3_/SnSe_2_ heterostructure; SEM, TEM, HRTEM images, and EDX elemental mapping of **(B–D)** SnSe_2_ and **(E–G)** Bi_2_Se_3_/SnSe_2_ heterostructure.

The preparation of Bi_2_Se_3_/SnSe_2_ heterostructures involved a simple solvothermal method. Its synthesis process is shown in Figure 1A. Precisely, 0.2 mmol of the pre-synthesized SnSe_2_ was gently dispersed in 20 mL ethanol and stirred for 0.5 h. Subsequently, a specific quantity of Bi(NO_3_)_3_·5H_2_O was added to the suspension and vigorously stirred for another 0.5 h. The resulting mixture was then moved to a 25 mL Teflon-lined autoclave and the solvothermal reaction was performed at 180 °C for 12 h. Finally, the resulting product was collected and subjected to three washes with ethanol and water.

For simplicity, the Bi_2_Se_3_/SnSe_2_ heterostructures were labeled as BS-1, BS-2, and BS-5, reflecting the amounts of Bi salt added (0.020, 0.025, and 0.040 mmol) respectively. In contrast, the synthesis of pure Bi_2_Se_3_ involved the addition of 1 mmol Bi_2_O_3_, 3 mmol SeO_2_, and 0.4 g NaOH into 40 mL of ethylene glycol. After being vigorously stirred for 1 h, the resulting suspension was placed in a Teflon-lined autoclave and the reaction was performed at 180°C for 7 h. Subsequently, the resultant products were collected, subjected to multiple washes with ethanol and deionized water, and finally dried at 60°C for 10 h.

### 2.3 Characterization of materials

The morphologies of the samples were inspected using both FESEM (SIRION 200) and TEM (JEOL-1400). Elemental composition was characterized by EDX and elemental mapping, while crystal structure was analyzed by XRD measurements (Bruker D8 Advance). The chemical states were determined through XPS analysis using an ESCALAB 250 spectrometer.

### 2.4 Gas-sensing properties

The gas sensors were produced through applying a solution of sensing materials through drop-casting (10 mg/mL in ethanol) onto Ag-Pd interdigital electrodes on an alumina substrate measuring 6.6 × 6.0 mm. To evaluate the gas sensing performance, a homemade sensor-testing system was employed, and a simplified experimental setup is depicted in Supplementary Figure S1. The real-time changes in conductivity of the sensors was collected via the electrochemical workstation (CHI 630E). In the sensing experiments, a precise volume of test gas was injected into the 4 L test chamber via a syringe. The relative humidity levels were regulated using a CK-80G commercial humidity chamber by Kingjo. All tests were performed in room air at 25°C with a relative humidity of 40%–50%. The sensing response (*S*) was determined using the equation of *S* = (*R*_*g*_-*R*_*a*_)/*R*_*a*_, where *R*_*g*_ and *R*_*a*_ represented the sensor resistance in the target gas and in air, respectively. The response and recovery times represent the duration it takes for the sensor to reach 90% of the resistance change after a target gas was injected and released, respectively.

### 2.5 Manufacturing of flexible chemical sensor

The flexible chemical sensor, incorporating Bi_2_Se_3_/SnSe_2_ heterostructures, was intricately applied to a polyethylene terephthalate substrate with interdigital gold patterns. The fabrication procedure for the device is meticulous and mirrors a method similar to the one previously documented. (Wang et al., 2016; Wang et al., 2017).

## 3 Results and discussion

### 3.1 Material structure and morphology

Characterized by SEM, TEM, and HRTEM. Figures 1B, E display the SEM images of SnSe_2_ and BS-2 samples, respectively, and their insets are the corresponding TEM images. The SnSe_2_ exhibits nanoplates morphology with the uniform dimension of 500–800 nm. After the solvothermal process, the SnSe_2_ still retain its original shape, and the hexagonal Bi_2_Se_3_ nanoplates grow on the surface of SnSe_2_, forming a heterostructure with intimate contact. The HRTEM images in Figures 1C, F clearly demonstrate the presence of a heterojunction interface. The lattice spacings of 0.310 and 0.225 nm belong to the (002) and (012) planes of SnSe_2_, respectively, while the 0.360 nm spacing corresponds to the (101) plane of Bi_2_Se_3_. In addition, the EDX mapping results demonstrate the uniform distribution of Sn, Se, and Bi elements (Figures 1D, G), which further indicates the formation of interconnected structures between SnSe_2_ and Bi_2_Se_3_.

**FIGURE 2:**
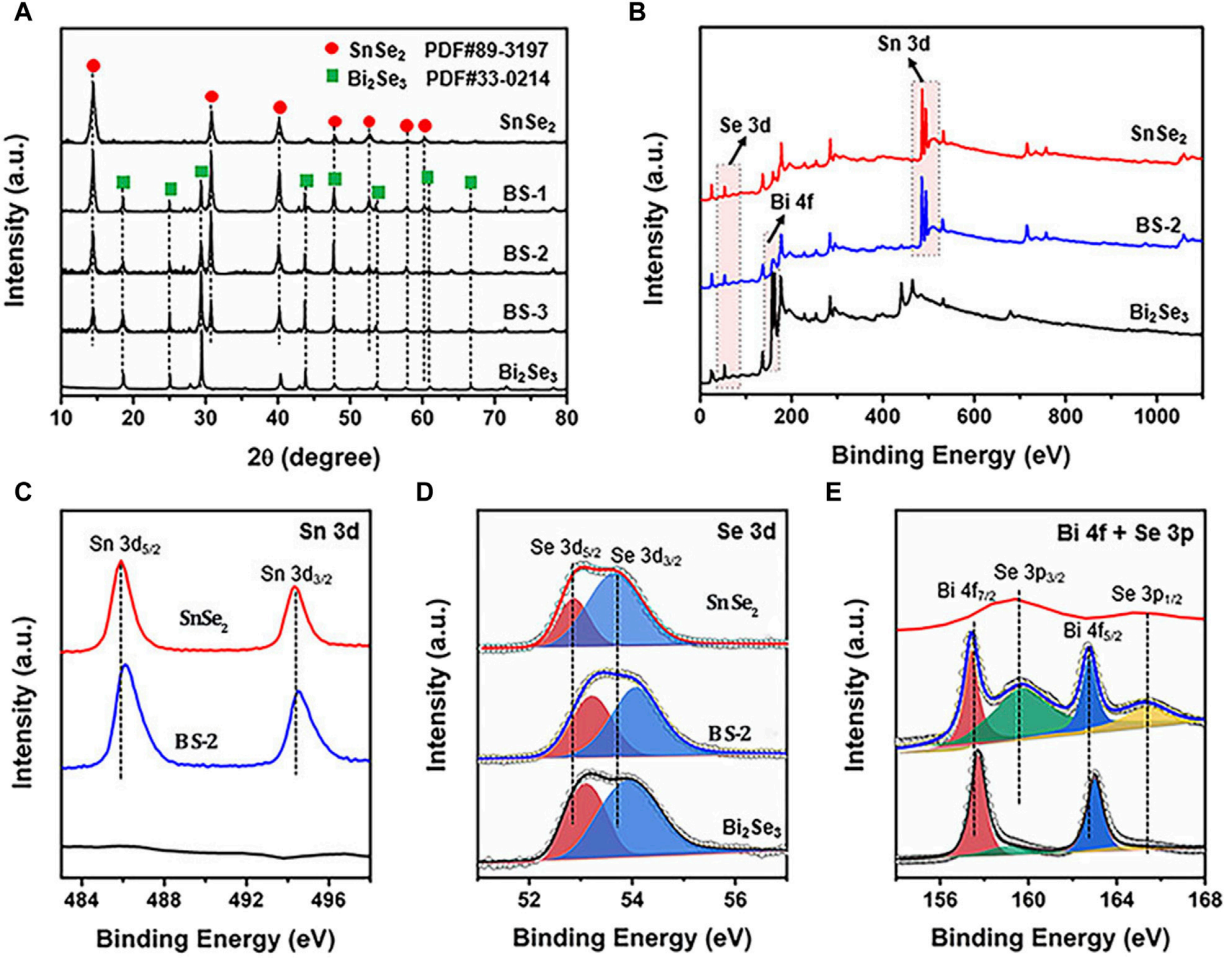
**(A)** XRD patterns of SnSe_2_, Bi_2_Se_3_, and SnSe_2_/Bi_2_Se_3_ heterostructure; XPS spectra of SnSe_2_, Bi_2_Se_3_ and BS-2 samples: **(B)** Survey, **(C)** Sn 3d, **(D)** Se 3d, and **(E)** Bi 4f and Se 3p.

A control sample of nanoplate-like Bi_2_Se_3_ was synthesized employing a solvothermal method for comparative analysis (Supplementary Figure S2). The crystalline characteristics of SnSe_2_, Bi_2_Se_3_, and the Bi_2_Se_3_/SnSe_2_ heterostructures were investigated via XRD analysis, with the resulting patterns depicted in Figure 2A. The diffraction peaks observed in the pristine samples were unambiguously assigned to hexagonal SnSe_2_ (JCPDS 89-3197) and hexagonal Bi_2_Se_3_ (JCPDS 33-0214). Specifically, the well-aligned distinct peaks at 14.41°, 30.73°, 40.08°, 47.69°, 52.57°, 57.81°, and 64.00° corresponded favorably to the (001), (011), (012), (110), (103), (201), and (202) crystallographic planes of SnSe_2_, respectively. Similarly, the peaks observed at 18.56°, 25.00°, 29.36°, 43.69°, 47.84°, and 53.54° were unequivocally attributed to the (006), (012), (015), (110), (116), and (205) crystallographic planes of Bi_2_Se_3_. With respect to the Bi_2_Se_3_/SnSe_2_ heterostructures, an increase in the amount of added Bi salt led to an augmentation in the diffraction peaks of Bi_2_Se_3_ and a simultaneous decrease in the peak assigned to SnSe_2_, without any noticeable impurities, thus indicating the high purity of these synthesized products.

XPS measurement was performed to analyze the chemical compositions and chemical states of the samples. The survey spectra of the BS-2 sample demonstrates the co-existence of Sn, Se, and Bi elements (Figure 2B), which is in line with the findings from the EDX mapping. In the detailed XPS spectrum of Sn3d, peaks at 485.9 and 494.3 eV are corresponding to Sn 3d_5/2_ and Sn 3d_3/2_ (Figure 2C), respectively, indicating the presence of Sn^4+^ in SnSe_2_. Two peaks around 52.8 and 53.6 eV belong to the 3d_5/2_ and 3d_3/2_ of Se^2-^ species (Figure 2D), respectively. In the Bi 4f and Se 3p spectra presented in Figure 2E, the distinctive peaks at 157.8 and 162.8 eV are indicative of Bi 4f_7/2_ and Bi 4f_5/2_, respectively, indicating the chemical state of Bi^3+^. The peaks that located at 159.7 and 165.4 eV could be separately assigned to the Se 3p_3/2_ and Se 3p_1/2_ orbitals of Se^2-^ (Xu et al., 2019). Notably, in comparison with pristine SnSe_2_, the binding energy of Sn 3d in BS-2 slightly shifts to higher energy, while the binding energy of Bi 4f in BS-2 are slightly lower than that of pure Bi_2_Se_3_. Such migration shifts of the binding energies may be ascribed to the change in electron density on the surfaces of the samples, and the above results confirm that the electrons in the heterostructure transfer from SnSe_2_ to Bi_2_Se_3_. The analyses conducted affirm the successful creation of Bi_2_Se_3_/SnSe_2_ heterostructures.

### 3.2 Gas sensing properties

**FIGURE 3:**
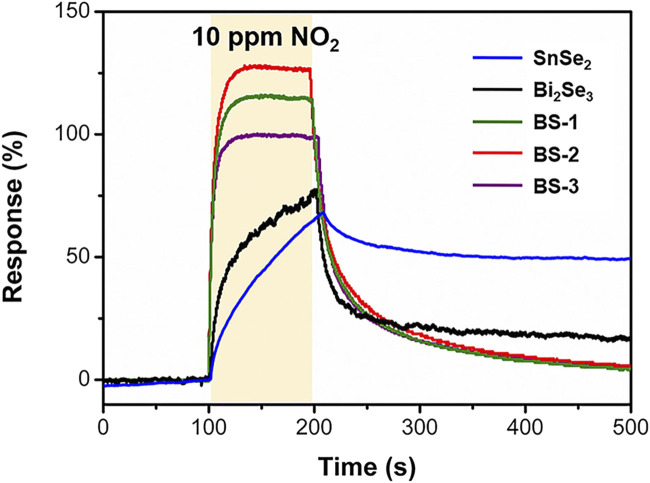
Response curves of the sensors based on SnSe_2_, Bi_2_Se_3_ and Bi_2_Se_3_/SnSe_2_ heterostructures to 10 ppm NO_2_ at room-temperature.

The NO_2_ sensing capabilities of Bi_2_Se_3_/SnSe_2_ heterostructures were examined through testing the change in resistance upon exposed to target gases. The initial investigation focused on assessing the influence of the Bi_2_Se_3_ to SnSe_2_ ratio on the sensing performance of the heterostructures. As the ratio of Bi_2_Se_3_ increased, the sensing response increases first then decreased (Figure 3). The BS-2 performs the most significant sensing response to NO_2_. By contrast, the sensors based on pure SnSe_2_ and Bi_2_Se_3_ exhibit inadequate response and sluggish recovery. The significantly improved sensing performance may be credited to the formed heterointerface between SnSe_2_ and Bi_2_Se_3_, which facilitates charge transfer and provides abundant active sites. According to the detailed sensing characteristics outlined in Supplementary Table S1, the Bi_2_Se_3_/SnSe_2_-2 sensor has been chosen for further research due to its impressive performance, showing the largest response rate of 130% and the shortest response/recovery times of 15/110 s to 10 ppm NO_2_.

**FIGURE 4:**
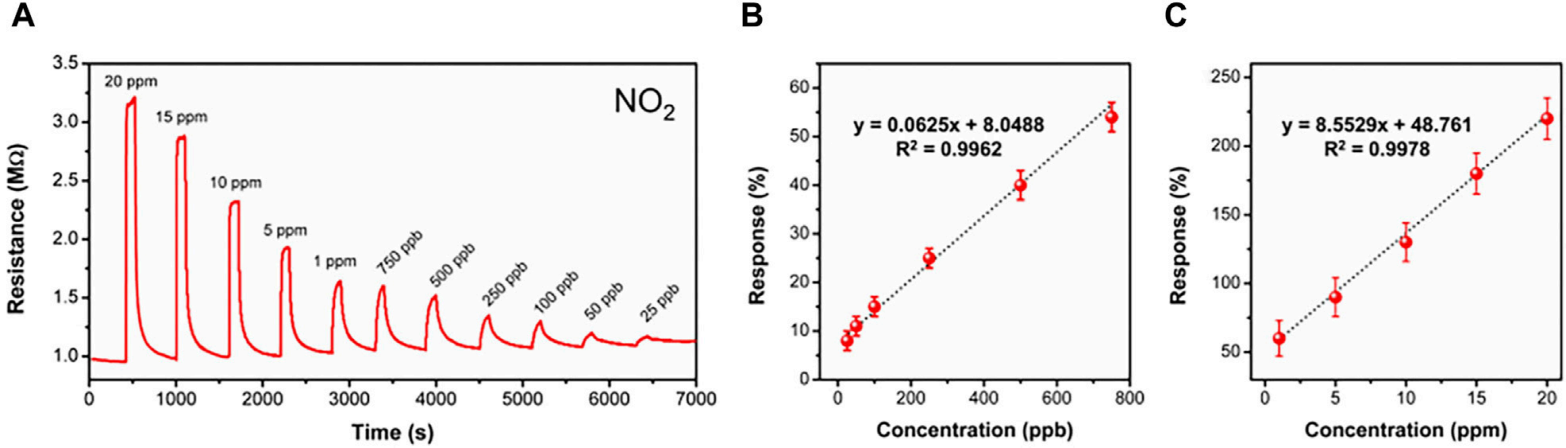
**(A)** Dynamic response curves of BS-2 sensor to NO_2_ (20 ppm–25 ppb); **(B, C)** The correlation between gas concentration and sensor response value.

The dynamic response curve of BS-2 sensor toward different NO_2_ concentration was further measured. As illustrated in Figure 4A, the sensor shows excellent response and recover ability, and the lowest detection limit for NO_2_ reaches 25 ppb. Besides, the correlation equation relating response value to gas concentration at ppb (Figure 4B) and ppm level (Figure 4C) could be separately calculated as a good linear relationship, which suggests its promising potential for sensor calibration purpose. Additionally, the wide detection range of the BS-2 sensor enables it to have a broad range of applications.

**FIGURE 5:**
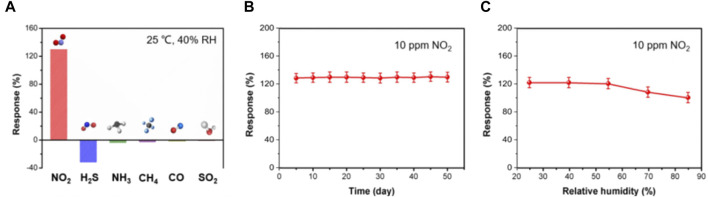
**(A)** Selective responses of BS-2 sensor to 10 ppm NO_2_, H_2_S, NH_3_, CH_4_, CO and SO_2_; **(B)** Long-term stability and **(C)** effects of humidity on the sensing responses of BS-2 sensor to 10 ppm NO_2_.

The selectivity of the BS-2 sensor was studied upon exposure to different analytes, including 10 ppm NO_2_, H_2_S, NH_3_, CH_4_, CO, and SO_2_. As shown in Figure 5A, the sensor demonstrates significantly larger response to NO_2_ than to other interfering gases, demonstrating its outstanding selectivity. To assess long-term stability, the responses of the BS-2 sensor to 10 ppm NO_2_ were recorded at a 5-day interval. The sensor exhibits a slight response recession (Figure 5B) and nearly identical sensing behaviors toward target gases within 50 days (Supplementary Figure S3), thereby affirming its significant stability and reliability. In addition, the BS-2 sensor presents notable stability when working in moderate humidity levels (25%–55%), making it suitable for practical applications (Figure 5C).

For a comprehensive assessment of sensing ability, Table 1 sums up a comparison between the optimal sensor in this study and other reported sensors based on 2D materials. The Bi_2_Se_3_/SnSe_2_ sensor shows superior NO_2_ sensing performance, featuring minimal power usage and heightened sensitivity, and notable response/recovery characteristics.

**TABLE 1:** Comparison of NO_2_ responses of Bi_2_Se_3_/SnSe_2_ sensor with that of other materials reported in literature.

Sensing materials	Working temperature/°C	Response (%)/concentration (ppm)	Detection limit/ppb	T_res_/T_rec_ (s)	References
MoS_2_ nanosheets	25	24.8/10	1,000	50/234	Liu et al. (2020)
trilayer WSe_2_	25	<64/10	/	-/-	Guo et al. (2019)
WSe_2_/WS_2_	25	66/10	—	-/1,500	Kim et al. (2020)
WSe_2_/MoS_2_	25	25/500	—	-/-	Kim et al. (2020)
This work	25	230/10	25	15/110	

**FIGURE 6:**
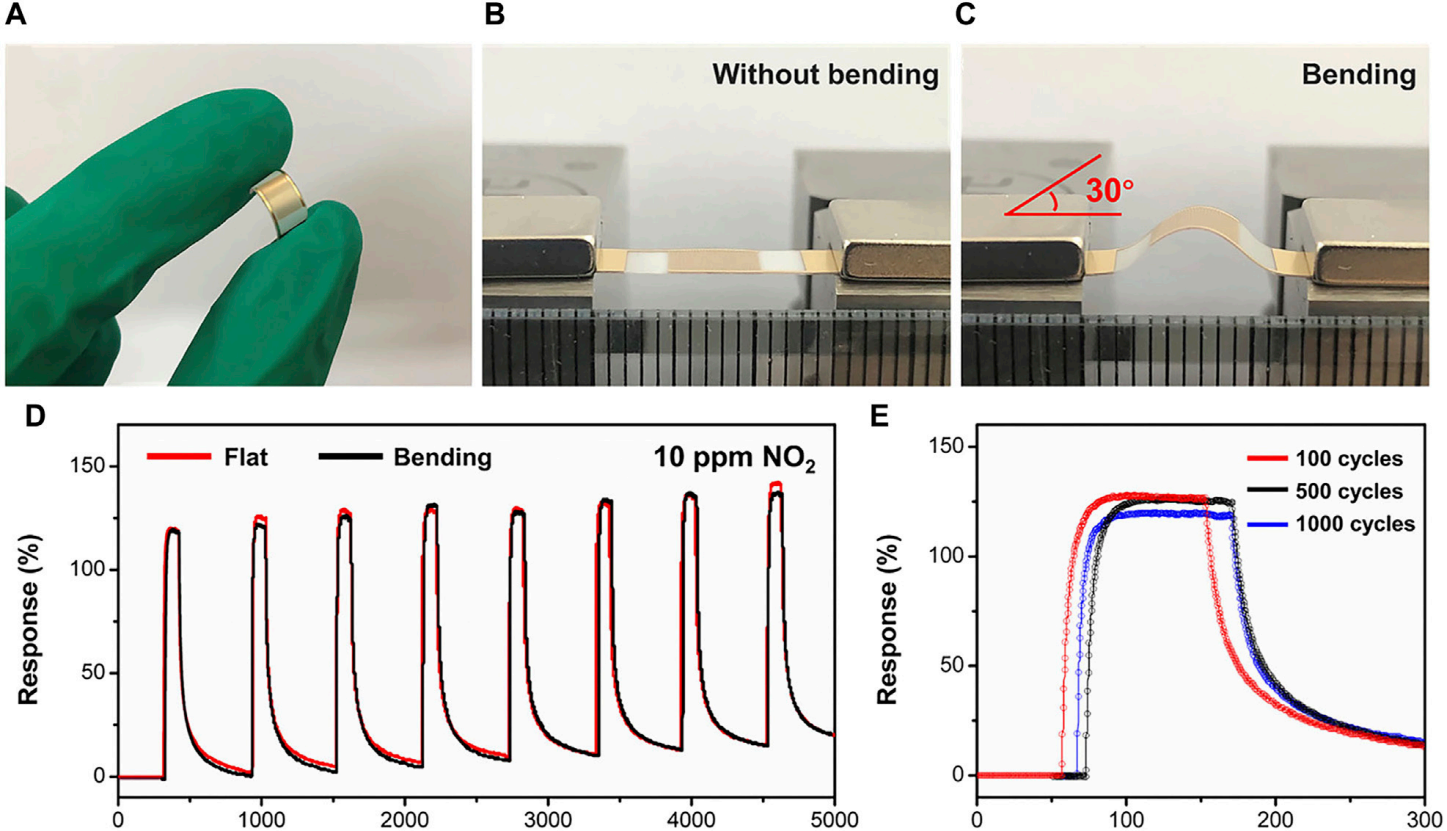
**(A–C)** Optical images of the flexible gas sensing device before and after bending; **(D)** Response/recovery curves of the flexible sensor without and with bending to 10 ppm NO_2_; **(E)** Gas sensing stability measured after 100, 500, and 1,000 bending cycles for NO_2_.

The sensor based on Bi_2_Se_3_/SnSe_2_ demonstrates outstanding NO_2_ sensing performance, showcasing minimal power usage and heightened sensitivity and remarkable response/recovery characteristics. To explore the potential capability, the mechanical flexibility properties of the BS-2 sensor were further studied. The flexible gas sensor was produced by depositing the Bi_2_Se_3_/SnSe_2_ heterostructures onto a PET substrate with gold interdigitated electrodes (Figures 6A–C). The gas sensing characteristics of the sensor under bending angle of 30° were then determined. As displays in Figure 6D the dynamic response and recovery curves in each trial are consistent with the flat condition. Furthermore, the fatigue test of the sensor after 100, 500, and 1,000 cycles bending and relaxing processes shows no significant degradation in response values (Figure 6E). The results presented above showcase the promising application potential of the flexible Bi_2_Se_3_/SnSe_2_ sensor in wearable sensing devices.

### 3.3 Gas sensing mechanism

**FIGURE 7:**
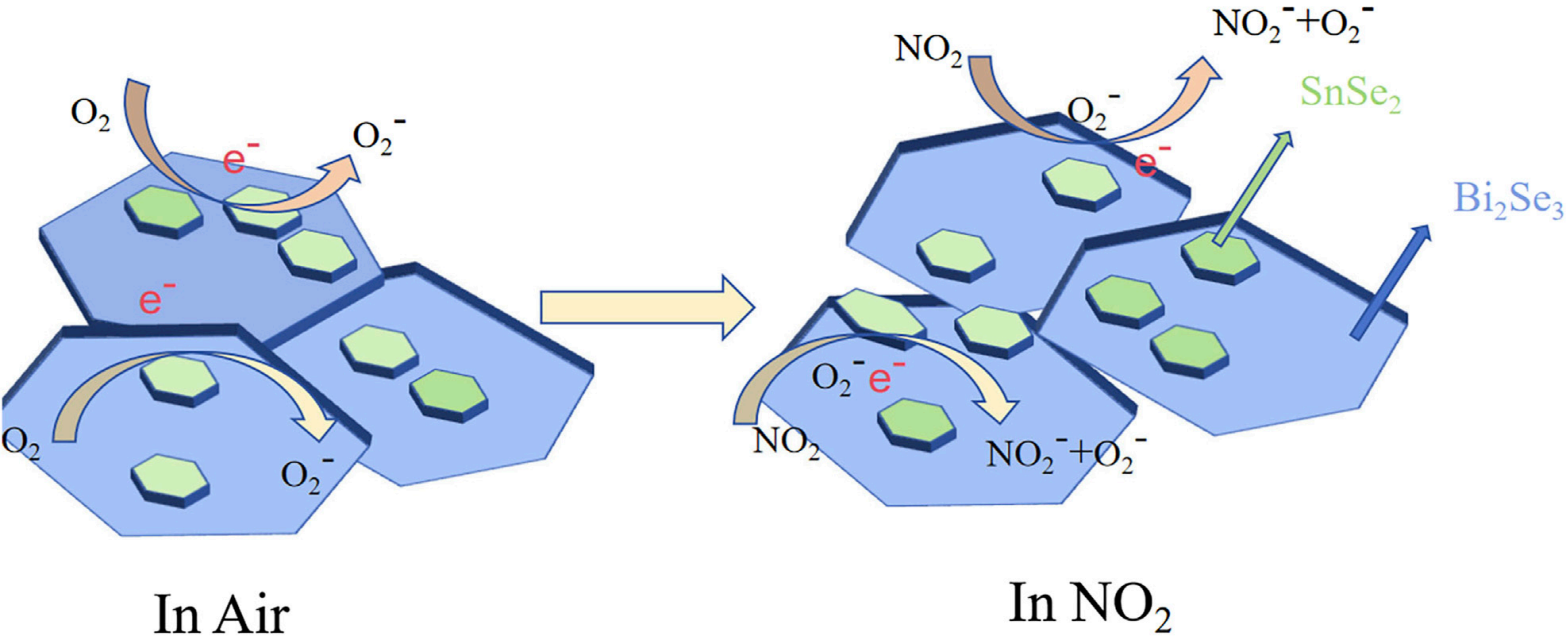
The gas sensing mechanism diagrams of Bi_2_Se_3_/SnSe_2_ sensors.

The gas sensing mechanism of semiconductive materials is established on the modification of resistance caused by the interaction between gas molecules and the sensing materials. (Zappa et al., 2018; Bag and Lee, 2019). The sensing mechanism of SnSe_2_ has been extensively discussed in the literature. As depicted in Figure 7, when exposed to air, O_2_ molecules adhere to the surface of n-type SnSe_2_, resulting in the creation of O^2−^ (ads) by capturing free electrons from the conduction band of SnSe_2_. At temperatures below 100°C, the main form of adsorbed oxygen is O^2−^.The redox reaction described above is illustrated in Eq. 1. (Kim et al., 2017).

$$O_{2}\left(\text{gas}\right)+e^{-}\;\to O^{2-}\left(\text{ads}\right)$$ (1)

When SnSe_2_ is exposed to NO_2_, the gas molecules can efficiently capture electrons from the conduction band of SnSe_2_ owing to the greater electronegativity of NO_2_ in comparison to O_2_. Furthermore, the reaction process can be delineated by Eqs 2, 3 below. Nevertheless, unmodified SnSe_2_ demonstrates a diminished response when compared to Bi_2_Se_3_/SnSe_2_. This phenomenon arises from the relatively low coverage of chemisorbed oxygen on the pristine SnSe_2_ surface. Consequently, NO_2_ can directly withdraw electrons from SnSe_2_. (Li et al., 2020). As a result, physical adsorption (Eq. 3) predominantly influences the sensing process.

$${NO}_{2}\left(\text{gas}\right)+e^{-}\to NO_{2}^{-}\left(ads\right)$$ (2)

$${NO}_{2}\left(\text{gas}\right)+O_{2}^{-}\left(ads\right)+{2e}^{-}\to {NO}_{2}^{-}\left(ads\right)+2O^{-}(ads)$$ (3)

**FIGURE 8:**
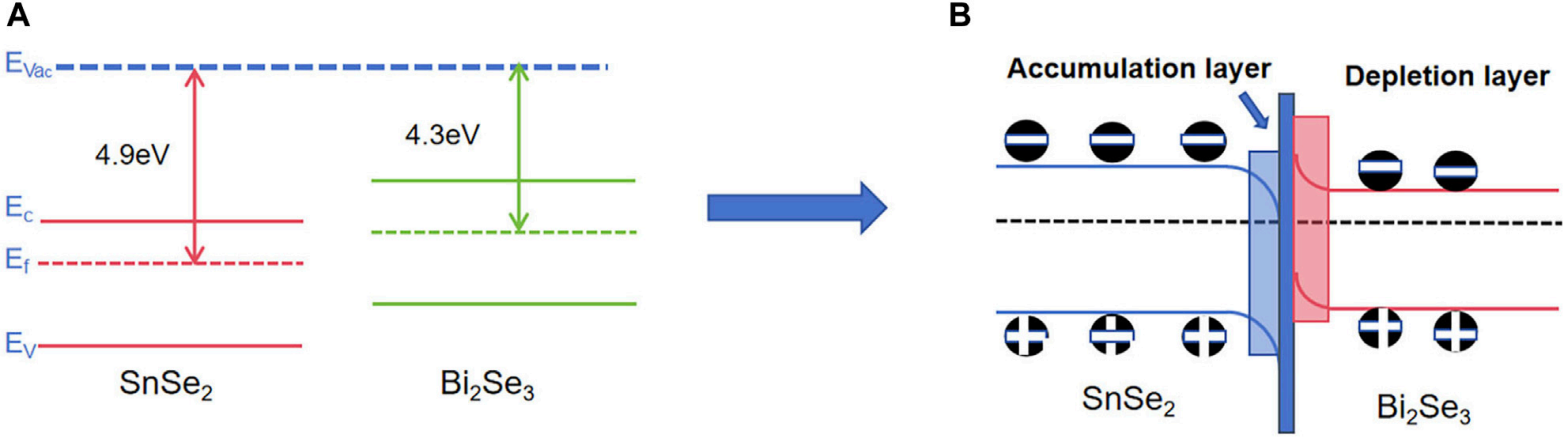
Schematic energy band diagram of SnSe_2_/Bi_2_Se_3_ heterostructure **(A)** before equilibrium; **(B)** After balancing.

The superior NO_2_ sensing properties of Bi_2_Se_3_/SnSe_2_ heterostructures are primarily attributed to the following factors building upon the foundational sensing mechanism discussed earlier:

Initially, the created n–n heterojunction between Bi_2_Se_3_ and SnSe_2_ plays a vital part in boosting the sensing response of the heterostructures. The electronic effects resulting from the construction of the SnSe_2_/Bi_2_Se_3_ heterojunction heterostructure are primarily attributed to band alignment. As depicted in Figure 8, the work function of Bi_2_Se_3_ is 4.3 eV, while the work function of SnSe_2_ is 4.9 eV. Therefore, the Fermi level of Bi_2_Se_3_ is notably higher than that of SnSe_2_. Consequently, upon the formation of the heterojunction, electrons transfer from Bi_2_Se_3_ to SnSe_2_, and holes transfer from SnSe_2_ to Bi_2_Se_3_, leading to the separation of holes and electrons. This results in the generation of an electron accumulation layer on the SnSe_2_ side and an electron depletion layer on the Bi_2_Se_3_ side, leading to electron accumulation on one side of SnSe_2_ and an improvement in the electronic structure at the interface.

During the sensing process of NO_2_ gas-sensitive materials, when NO_2_ gas molecules come into contact with the material surface in a detection gas environment, the gas is first adsorbed by the material. Due to the oxidizing nature of NO_2_, it then captures electrons from the surface of material, resulting in a change in the carrier concentration and leading to variations in resistance and circuit current. The sensing process can be divided into two primary steps: gas adsorption, where active sites on the material surface adsorb gas, and electron reaction, where gas interacts with electrons on the material surface. (Gong et al., 2019).

In the 2D-2D SnSe_2_/Bi_2_Se_3_ heterostructure material, where the sensing material remains SnSe_2_, the aggregation of electrons on the surface of SnSe_2_ after heterostructure equilibrium is achieved enhances the interaction between NO_2_ gas and the material. This leads to a larger change in resistance signal, thereby improving the sensitivity of the material to NO_2_ gas. The synthesized Bi_2_Se_3_/SnSe_2_ 2D/2D heterostructure is lamellae-stacked, which has relatively more adsorption sites and large specific surface area, which is conducive to the adsorption and desorption of NO_2_. Moreover, the heterostructure exhibits significantly reduced response and recovery time duing to the enhanced electron transfer rate within the material.

## 4 Conclusion

In summary, Bi_2_Se_3_/SnSe_2_ 2D/2D heterostructures are successfully synthesized and used for NO_2_ detection. The optimized Bi_2_Se_3_/SnSe_2_ heterostructure exhibited rapid response towards NO_2_ gas. Compared to pure SnSe_2_, the response time was significantly reduced from 73 to 15 s (10 ppm). The enhanced sensing performance is a direct result of the abundant n-n heterojunctions, improved interface charge transfer, and increased active sites that are inherent in the SnSe_2_/Bi_2_Se_3_ heterostructure. Furthermore, the sensor displayed excellent selectivity, with a low detection limit of 10 ppb and a broad detection range from 10 ppb to 20 ppm. Furthermore, the fatigue test of the sensor after 100, 500, and 1,000 cycles bending and relaxing processes shows no significant degradation in response values. These results offer important insights for selecting materials and designing heterostructures to achieve effective room temperature gas detection in a range of applications.

## Data Availability

The original contributions presented in the study are included in the article/Supplementary Material, further inquiries can be directed to the corresponding authors.
