# Supplementary material for: 2D/2D Bi2Se3/SnSe2 heterostructure with rapid NO2 gas detection
Source: Front Chem. 2024 Jul 26;12:1425693. doi: 10.3389/fchem.2024.1425693 (PMC11309994; doi:10.3389/fchem.2024.1425693)
Supplement: Supplementary file 1 [file DataSheet1.docx]

Supplementary Material

# Supplementary Figures


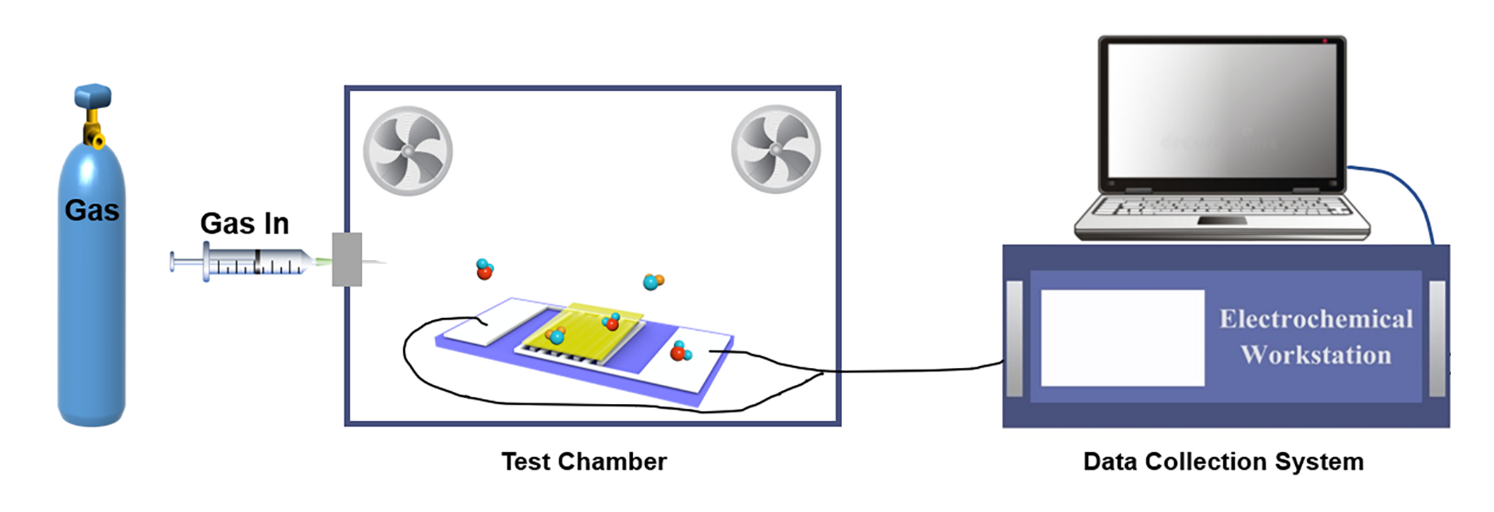


Figure S1 Schematic diagram of the sensor measurement.

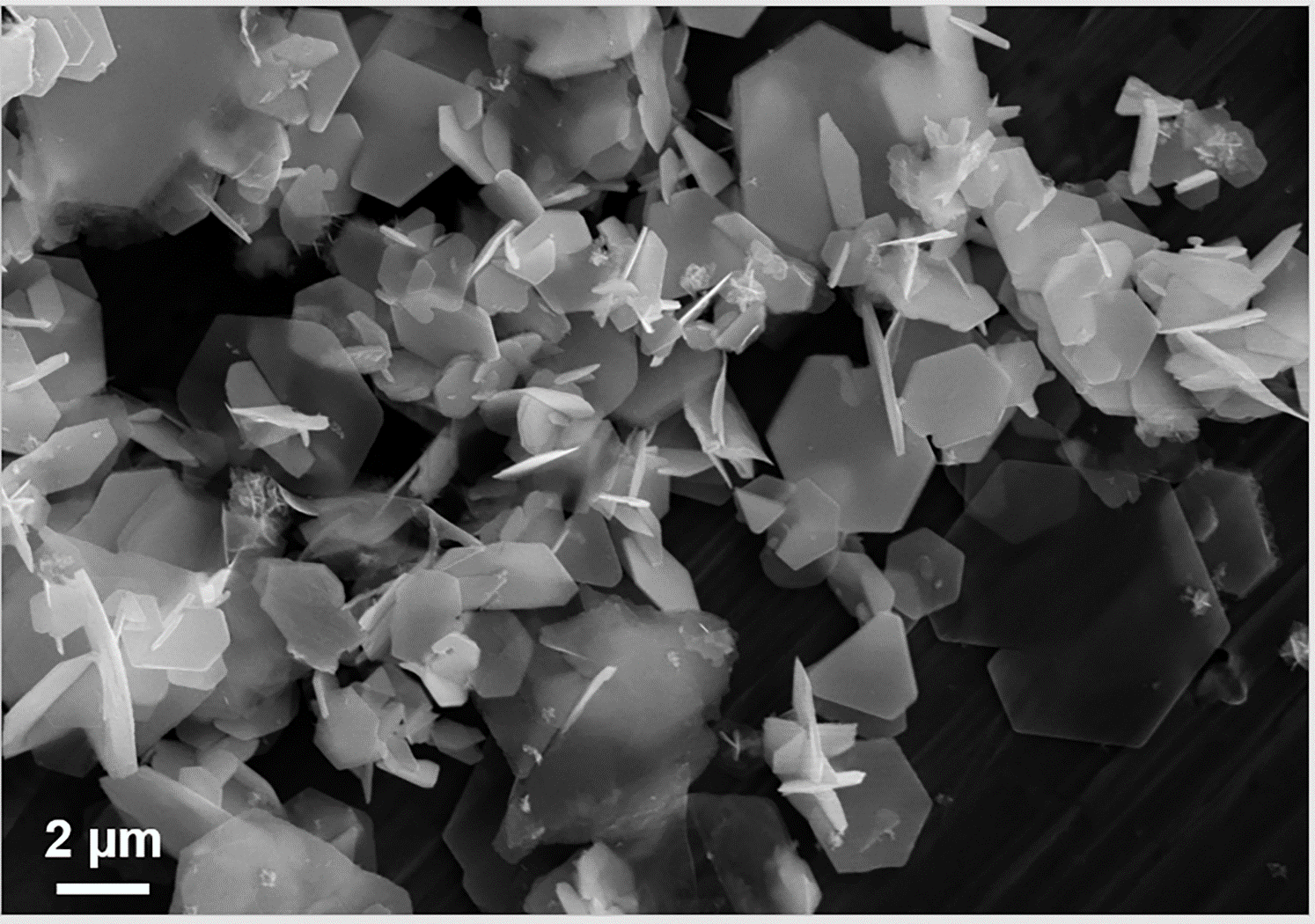
Figure S2 SEM image of Bi_2_Se_3_.


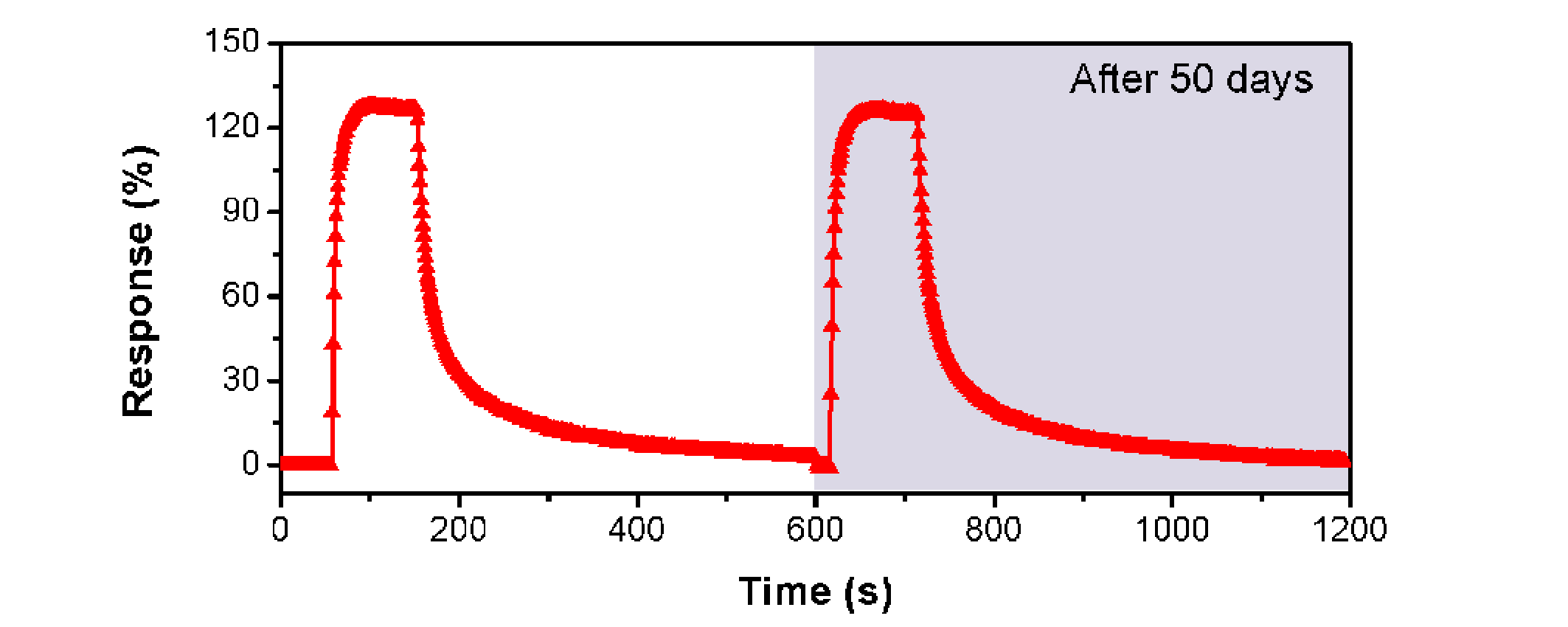


Figure S3 Long-term stability of the BS-2 sensor to10 ppm NO_2_ after 50 day

# Supplementary Table

**Table S1** Response value and response/recovery time of SnSe_2_, Bi_2_Se_3_ and Bi_2_Se_3_/SnSe_2_ sensors toward 10 ppm NO_2_.

| Sensing materials | 10 ppm NO_2_ | |
| --- | --- | --- |
|  | Response (%) | t_res_/t_rec_ (s/s) |
| SnSe_2_ | 65 | 73/>300 |
| Bi_2_Se_3_ | 76 | 70/200 |
| BS-1 | 114 | 14/112 |
| BS-2 | 130 | 15/110 |
| BS-3 | 98 | 13/108 |
